# Sex Hormones, Growth Hormone, and the Cornea

**DOI:** 10.3390/cells11020224

**Published:** 2022-01-11

**Authors:** Tina B. McKay, Shrestha Priyadarsini, Dimitrios Karamichos

**Affiliations:** 1 Department of Cell Biology, University of Oklahoma Health Sciences, Oklahoma City, OK 73104, USA; tmckay@mgh.harvard.edu; 2 Dean McGee Eye Institute, Oklahoma City, OK 73104, USA; shrestha.priyadarsini@gmail.com; 3 North Texas Eye Research Institute, University of North Texas Health Science Center, Fort Worth, TX 76107, USA; 4 Department of Pharmaceutical Sciences, University of North Texas Health Science Center, Fort Worth, TX 76107, USA; 5 Department of Pharmacology and Neuroscience, University of North Texas Health Science Center, Fort Worth, TX 76107, USA

**Keywords:** cornea, estrogen, estradiol, growth hormone, insulin growth factor-1, wound healing

## Abstract

The growth and maintenance of nearly every tissue in the body is influenced by systemic hormones during embryonic development through puberty and into adulthood. Of the ~130 different hormones expressed in the human body, steroid hormones and peptide hormones are highly abundant in circulation and are known to regulate anabolic processes and wound healing in a tissue-dependent manner. Of interest, differential levels of sex hormones have been associated with ocular pathologies, including dry eye disease and keratoconus. In this review, we discuss key studies that have revealed a role for androgens and estrogens in the cornea with focus on ocular surface homeostasis, wound healing, and stromal thickness. We also review studies of human growth hormone and insulin growth factor-1 in influencing ocular growth and epithelial regeneration. While it is unclear if endogenous hormones contribute to differential corneal wound healing in common animal models, the abundance of evidence suggests that systemic hormone levels, as a function of age, should be considered as an experimental variable in studies of corneal health and disease.

## 1. Introduction

The cornea is an avascular, immune-privileged, transparent tissue located at the front of the eye that protects the posterior segments from infection and injury. The structure of the cornea is composed by three primary cell layers: (1) a non-keratinized, stratified epithelium, (2) keratocytes dispersed in a lamellar hydrated collagen matrix, and (3) a single layer of endothelium that regulates fluid flux from the aqueous humor. Two acellular layers known as Bowman’s layer and Descemet’s membrane serve to separate the stroma from the epithelium and endothelial layers, respectively. Within the stroma and epithelium, sensory nerves originating from the trigeminal ganglion densely innervate the cornea in a centripetal pattern allowing for close monitoring of ocular surface temperature and osmolarity. An overlaying tear film on the surface of the cornea is essential for providing dissolved oxygen and nutrients to the corneal epithelium and lubrication of the ocular surface, while maintaining an anti-microbial environment with the presence of lysozyme and other proteins that inhibit bacterial and fungal growth (reviewed in Ref. [1]). The tear film also functions as a conduit for systemic factors, including metabolites and hormones, to reach the corneal surface.

Hormones are signaling molecules that are secreted by select cells and may induce biological effects in cells that express the respective receptor. There is significant overlap between classification of the different hormones found in the human body and likewise a moderate complexity in their temporal regulation during development and aging. Hormones have been sub-divided based on their chemical derivation (steroid, amino acid, peptide, protein, glycoprotein, or eicosanoid), mechanism of uptake (lipophilic hormones that diffuse through the plasma membrane versus surface-binding hormones), and functional effects (metabolic—insulin and glucagon; morphogenetic—growth hormone, luteinizing hormone, thyroid hormone; or kinetic—epinephrine). Hormones may also be classified based on their local or distal effects or stimulation patterns that are mediated via the secretion of other hormones (tropic versus non-tropic). Moreover, hormones may selectively target endocrine glands or bind to non-endocrine tissues in a cell-specific manner leading to the activation of downstream effectors that initiate a wide-ranging, physiological response.

The effects of androgens and estrogens (both cholesterol-derived) and growth hormone (GH, peptide) on tissue growth and regeneration have been most-well characterized due to their high systemic abundance and targeted effects associated with aging, and thus we focus our review of the literature on these select hormones. Insulin growth factor-1 (IGF-1) is an important mediator involved in the pituitary GH-axis with known GH-independent properties that differentially affect a number of tissues within the body, including the cornea. Far less is known regarding other hormones found in circulation in the context of corneal biology and remains an area for further exploration. The role of hormones in the corneal-thinning disease, keratoconus, has been recently reviewed [2,3,4,5]. Here, we focus on the broad function of hormones in corneal physiology with a review of the sex hormones (androgens and estrogens) and growth-associated hormones, GH and IGF-1, and their reported roles in ocular surface maintenance, wound healing, and tissue growth.

## 2. Androgens and Estrogens

The biosynthesis of androgens and estrogens occurs in the testes and ovaries, with small contributions from the adrenal gland, and requires cholesterol in a highly regulated and tissue-dependent manner. As the primary precursor involved in steroidogenesis, cholesterol can be provided to a cell via in situ synthesis, cellular uptake from circulation, or by mobilization of cholesteryl ester stores [6]. Many of the enzymes required for steroid biosynthesis, such as cytochrome P450scc, ferredoxin reductase, and 3β-hydroxysteroid dehydrogenase, are localized to the mitochondria, where the biosynthesis of steroids is initiated [7,8,9]. Mitochondrial transport of cholesterol from lipid droplets involves SNARE proteins (soluble NSF attachment receptor), which are required for hormone synthesis in steroidogenic tissues [10,11]. Diseases associated with an inability to synthesize steroids, such as congenital lipid adrenal hyperplasia, are linked with mutations in the enzyme required for cholesterol transport into the mitochondria, including the steroidogenic acute regulatory protein ([12] and reviewed in Ref. [13]).

**Figure 1:**
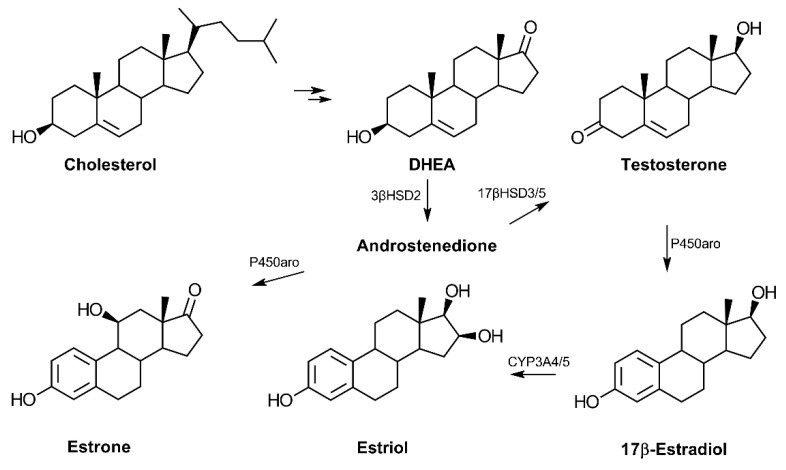
Biosynthesis of androgens and estrogens from cholesterol. Cholesterol is converted to pregnenolone and then 17-hydroxypregnenolone before synthesis of dehydroepiandrosterone (DHEA). The common precursor, androstenedione, can be converted to the primary estrogens or testosterone in a tissue-dependent manner. The adrenal cortex is the primary site of C19-hormone synthesis from cholesterol in humans. Estrone may also be converted to estradiol via the enzyme 17β-hydroxysteroid dehydrogenase type 1 (17βHSD1) (*not shown*). (Abbreviations: 3β-hydroxysteroid dehydrogenase type 2 (3βHSD2), 17β-hydroxysteroid dehydrogenase type 3 or 5 (17βHSD3/5), cytochrome P450 aromatase (P450aro), and cytochrome P450 3A4 (CYP3A4)).

In terms of biosynthesis, the conversion of cholesterol to pregnenolone is the first step in the production of dehydroepiandrosterone (DHEA) (reviewed in Ref. [14]) (Figure 1). Following cellular uptake, systemic DHEA-sulfate (DHEA-S) is converted into the non-sulfated DHEA, which is then metabolized to other hormones depending on the tissue. DHEA serves as a common precursor to androgens and estrogens via the production of androstenedione, which can then be converted to testosterone. Testosterone is an androgen that can be produced from DHEA in the testes and prostate and may serve as a precursor to the estrogens. In addition to its role in promoting features associated with masculinity, testosterone has also been reported to influence cognitive function in an age-dependent fashion [15]. DHEA is an established source for the biosynthesis of the three primary estrogens: estrone, 17β-estradiol, and estriol. The primary estrogen receptors (ER), ERα and ERβ, have genes that are present on chromosome 14, while the androgen receptor gene is localized on the X chromosome [16,17].

In humans, DHEA serum levels increase during adrenarche with continual increases from birth to 18.5 years of age in both males and females [18]. The sulfated form of DHEA, DHEA-S, is the most highly concentrated hormone in circulation in humans but appears largely absent in rodents [19,20]. Likewise, the enzyme responsible for DHEA synthesis, 17α-hydroxylase, is not expressed in the mouse or rat adrenal cortex, signifying significant species-related differences in regards to DHEA synthesis and likely function [19].

## 3. Sex Hormones and Their Receptors in the Cornea

Both androgens and estrogens are present in the aqueous and vitreous humors of the adult eye. Testosterone, progesterone, estrone, and 17β-estradiol have been detected at sub-nanogram per milliliter concentrations in the aqueous and vitreous humor in the bovine eye [21,22]. The derivation of these hormones in the cornea is likely from circulation, either via the aqueous humor or the overlaying tear film. Moreover, tissues of the anterior surface, cornea, conjunctiva, meibomian, and lacrimal glands also appear to express the enzymes required for endogenous hormone production from cholesterol, including aromatase, glucuronyltransferase, and 3β-hydroxysteroid dehydrogenase isomerase type 1 [23]. Autocrine or paracrine production of hormones may also be contributing to the presence of hormones found in the anterior segment.

Though the function of hormones in the cornea remains unclear, reports have suggested that the receptors for androgens and estrogens, among others, are expressed (at the gene and protein levels) by corneal epithelial and stromal fibroblasts (Table 1). A study by Suzuki et al. reported the identification of estrogen receptors (ERα, ERβ) and progesterone receptor in immunohistology stains of the human cornea showing protein expression in the epithelial, stroma, and endothelial layers in both male and female human corneas, suggesting that the cornea has the capacity to be responsive to systemic levels of androgens and estrogens [24]. These studies provide supporting evidence that sex hormone receptors may be expressed by the human cornea. Other relevant hormones including luteinizing hormone receptor and follicle-stimulating hormone receptor are also expressed by human corneal stromal fibroblasts [25]. It is likely that events that affect systemic estrogen levels, such as menopause or hormone therapy, may influence the corneal stroma.

### 3.1. Maintenance of the Ocular Surface

Consistent with an important functional role for hormones in maintaining corneal homeostasis, deficits in androgens and estrogens may have negative effects on ocular surface health primarily as a function of decreased tear production associated with aging. Sex-dependent effects of hormones on immune cells and estrogen-mediated immunoregulation have been thoroughly described and reviewed [29,30,31,32,33]. Androgens are known to influence the expression and secretion of lipids from acinar epithelial cells found in the meibomian gland, the main source of the lipid layer of the tear film [34]. In general, androgen replacement therapy appears to remedy many of the detrimental ocular effects of hormone insufficiency by restoring adequate tear production and quality to maintain lubrication of the corneal surface. Studies testing the effects of supplementation with dihydrotestosterone (DHT), a hormone derived from testosterone, by ocular application or subcutaneous injection in male BALB/c mice that had undergone orchiectomy have shown moderate rescue of the ocular phenotype exhibited by androgen insufficiency, including epithelial defects and decreased tear production [35]. Moreover, ocular application of DHT significantly increased detectable serum levels compared to castrated mice correlating with a moderate absorption of the androgen through the cornea and into circulation, even though only the subcutaneously injected group showed adequate recovery of tear function [35].

Similar positive effects of hormone supplementation have been studied in small human clinical trials of ocular surface health and tear osmolarity and production in older women following menopause [36]. A large cohort study (*n* = 3968) also identified lower cataract development (12%) and slightly higher pterygia (7%) in women who were supplemented with estrogens [37]. In animal models, the effects of estrogen deficiency on reduced tear film production and increased prevalence of epithelial defects have been reported in female rats that had undergone ovariectomy [38]. Removal of the ovaries resulted in a significant reduction in 17β-estradiol levels following surgery (~two-fold, *p* < 0.01) [38]. Lower 17β-estradiol levels also correlated with an increase in matrix metalloproteinase-2 expression in the lacrimal glands in this study, as testosterone treatment appeared to reverse these effects, supporting a role for androgen-insufficiency, rather than a sole lack of estrogen, in mediating the observed effects on the ocular surface [38]. Given the known function of systemic androgens as precursors to estrogens and other androgens in a tissue-specific manner, it is possible that androgen supplementation contributes to adequate estrogen production required for maintaining the integrity of the ocular surface.

In terms of systemic conditions that contribute to significant ocular pathologies, autoimmune diseases are known to occur with sexual dimorphism, as females comprise roughly 80–90% of the autoimmune patient population (reviewed in Ref. [39]). Dry eye is a major co-morbidity of many autoimmune diseases, such as Sjögren syndrome and systemic lupus erythematosus [40]. The non-obese diabetic (NOD) mouse models, such as NOD.B10.H2^b^, develop symptoms like those found in patients with Sjögren syndrome with the presence of increased pro-inflammatory cytokines and production of autoantibodies in circulation. Ovariectomy procedures in these mice have been reported to result in an acceleration of the inflammatory condition and are characterized by defects in lacrimal gland function [41]. In evaluating the effects of estrogen deficiency on lacrimal gland structure and function, a study by Rahimi et al. utilized a knock-out mouse model lacking aromatase, the primary enzyme responsible for estrogen synthesis, thereby eliminating in situ estrogen production [42]. They found little effect on the transcription of genes associated with inflammation and no clinical differences in hematoxylin and eosin-stained lacrimal glands in aromatase knock-out mice compared to wild-type [42]. These results suggest that a lack of estrogen production alone is not sufficient to promote lacrimal gland dysfunction as that associated with the human condition. It is possible that the inflammatory processes that are found in Sjögren syndrome animal models lacking the ability to synthesize estrogen may require a genetic background with elevated cytokine and/or autoantibody production in order to give rise to the observed pathologies associated with ocular surface damage and lacrimal gland dysfunction.

Interestingly, androgens are thought to give rise to an anti-inflammatory response that may serve to protect the lacrimal gland from immune dysregulation and promote retention of tissue function [43]. While DHT stimulates a potent anti-inflammatory response in meibomian gland epithelial cells cultured in vitro, DHT does not appear to suppress pro-inflammatory genes in corneal epithelial cells [44]. In contrast, 17-estradiol application increased the expression of pro-inflammatory genes by corneal epithelial cells cultured in vitro [45], suggesting notable cell-specific differences in the functional regulatory properties of hormones and their effects on the eye.

### 3.2. Wound Healing

The extracellular matrix (ECM) of the corneal stroma is devised of a well-organized collagenous matrix containing proteoglycans, such as keratocan, decorin, lumican, and mimecan, which collectively support the structural, hydrous, and refractive properties of the cornea that allows for quality vision. The capacity of a tissue to repair damage is influenced by many factors, including the inflammatory response, epithelial proliferation, and ECM deposition. Hormones are known to influence all of these processes, which collectively contribute to sex-specific differences in the rate and effectiveness of wound healing in a tissue-dependent manner, as has been reported in humans [46] and rodents [47,48]. Prolonged stages of tissue repair can result in fibrosis, failed epithelial closure, and chronic inflammation leading to permanent tissue damage(s). These processes occur in various pathological conditions, such as diabetes and fibrotic conditions, which can promote the development of re-occurring infections, loss of tissue transparency, and visual debilitation.

**Figure 2:**
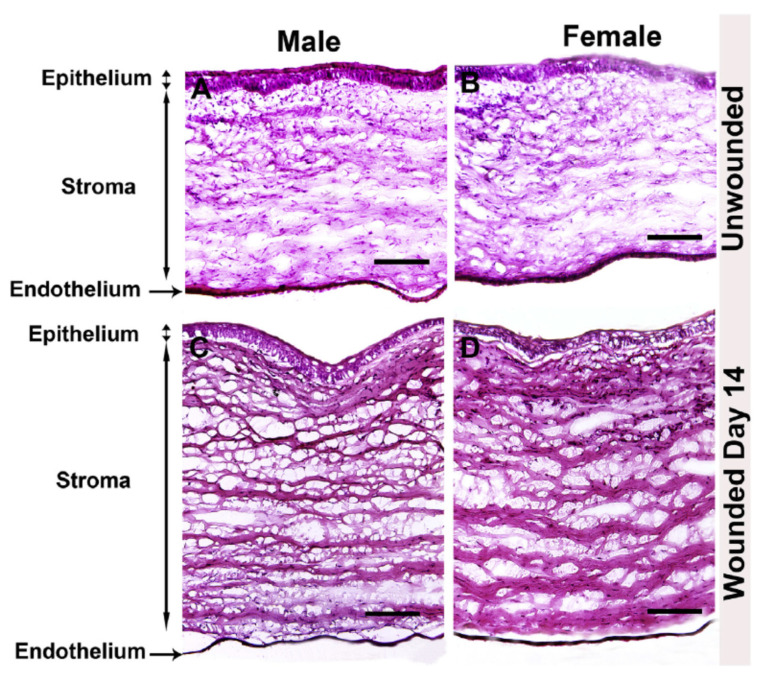
Comparative study of male and female rabbit corneas following wounding. Hematoxylin and eosin stain of unwounded and wounded (**A**,**C**) male and (**B**,**D**) female rabbit corneas. Image re-produced from [49] with permission (https://doi.org/10.1016/j.exer.2019.107705, accessed 13 December 2021).

Compared to wound healing within skin, corneal wound healing requires precise ECM secretion, deposition, and organization to preserve the clarity and transparency of the tissue. The formation of myofibroblasts by resident keratocytes mediates rapid deposition of collagen and other ECM proteins to enable closure of the wound, as well as to recruit phagocytic inflammatory cells to remove cell debris. This remodeling process occurs in a relatively short period of time and must be controlled to prevent scarification of the corneal tissue. An important study evaluating male and female rabbits has been recently reported as a comparative analysis of scar development following alkaline burn (Figure 2). Neither gross morphological differences nor differences in α-smooth muscle actin, fibronectin, or collagen type I expression were reported as a function of sex [49]. To the authors’ knowledge, no evaluation of relative estrogen or androgen levels was performed in this study, which requires further investigations into the role of these factors in pro-fibrotic processes during tissue regeneration. However, this work provides evidence that no overt differences in corneal wound healing or stromal scar development as a function of sex are present in a well-characterized rabbit model [49].

While wound healing in the rabbit cornea appears to not be overtly influenced by sex [49], young BALB/c female mice have been reported to show slower regeneration of the corneal epithelium following debridement [50]. These findings appear similar in male mice exposed to exogenous 17β-estradiol applied to the cornea, suggesting that estrogen influences corneal epithelial regeneration independent of sex. The sex-dependent differences in wound healing identified in this study are largely attributed to targeted effects on immune cell populations that are absent in common in vitro systems. In conventional cultures of corneal epithelial cells, exogenous 17β-estradiol has been shown to increase epidermal growth factor (EGF) levels and promote corneal epithelial cell migration and proliferation in vitro [51]. The observed effects of estradiol on epithelial cells are likely a function of its regulation and promotion of EGF expression, which has previously been reported to be an important mediator of corneal wound healing via activation of nuclear factor κB (NFκB) p50 downstream pathways [52,53,54]. The mechanism by which 17β-estradiol increases EGF levels has also been found in other tissues, including the uterus, where gene transcription of the EGFR is induced in response to exogenous 17β-estradiol, as an ER-responsive element [55]. In a feedback loop between EGF and estrogen-mediated signaling, EGF may, in turn, activate ER-responsive genes following internalization and translocation of the ER to the nucleus [56], suggesting a dynamic interplay between estrogen hormones and pro-survival and -migratory mechanisms.

### 3.3. Corneal Stromal Thickness

**Figure 3:**
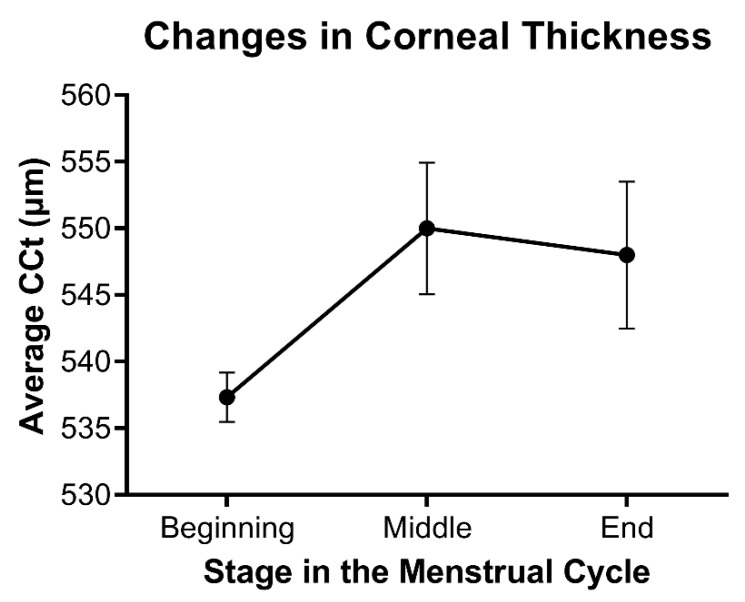
Relative changes in stromal thickness as a function of the menstrual cycle. Data based on the average central corneal thickness (CCT) reported at each stage in references [57,58,59]. Mean ± standard error shown, with *n* = 3.

Consistent with the effects of hormones during wound healing, differences in corneal thickness as a function of hormone flux have also been reported in multiple studies. Corneal thickness has been found to fluctuate during menstruation, pregnancy, and lactation, all of which are characterized by notable changes in hormone levels. The observed increase in corneal thickness during mid-ovulation of the menstrual cycle around day 14 and then again towards the end of the cycle correlates with a systemic increase in estrogen and progesterone, respectively (Figure 3) [57,58,59]. Studies evaluating the effects of estrogens on corneal biomechanics in an ex vivo model have found that topically-applied 17β-estradiol reduced corneal stiffness [60]. The topical application of 17β-estradiol in a rabbit model showed a reversible myopic shift independent of differences in corneal thickness [61]. Of note, water retention and fluid influx are influenced by estrogen levels and may also be a major regulator of corneal thickness in an independent manner to matrix deposition within the stroma.

Hormone levels may also contribute to clinical outcomes, as variations in corneal thickness may influence the result of laser refractive surgery [62,63]. Several risk factors have been correlated with corneal ectasia development post-LASIK, including *forme fruste* keratoconus (KC), pellucid marginal degeneration, and a central corneal thickness of less than 500 μm [62]. Furthermore, several studies have reported case studies of post-LASIK corneal ectasia developing following pregnancy, further supporting evidence that variations in the hormones levels that occur during pregnancy may influence corneal structure [64,65,66,67].

KC is a common corneal thinning disease that has attracted extensive attention in recent years. A sex bias has been reported in the KC population with higher male prevalence compared to females in a ratio of roughly 3:2 depending on the cohort [68,69]. Studies from our lab identified elevated salivary levels of DHEA-S and reduced estrone in the KC population compared to age- and sex-matched controls [70]. Moreover, a hormone primarily secreted by the thyroid, thyroxine, has been reported to be higher in KC tears compared to controls [71,72], with a single case study reporting acute KC occurring co-incidentally with hypothyroxinemia onset [73]. In agreement with an increased occurrence of post-LASIK corneal ectasia, multiple studies have found KC progression or onset to be influenced by pregnancy [74,75,76,77]. A case study also reported a small number of individuals who developed KC following in vitro fertilization treatment [78]. An important question within the field remains why corneal thinning stabilizes in the majority of KC patients by the age of 30–40 years old. The role of endocrine function in KC onset, progression, and stabilization remains unclear but likely involves a number of factors, including genetic, environmental, or epigenetic factors that contribute to altered keratocyte functionality in the secretion, assembly, and maintenance of collagen lamellae within the central corneal stroma.

## 4. Roles of Growth Hormone and Insulin-Like Growth Factor in the Cornea

**Figure 4:**
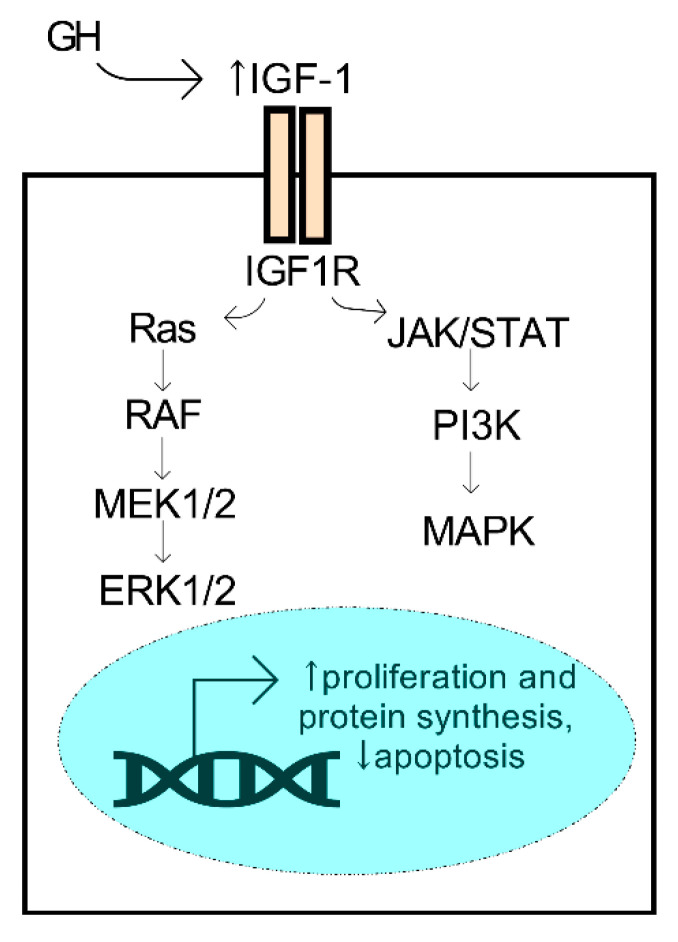
Activation of the GH/IGF-1 axis promotes pro-survival mechanisms. GH secreted from the pituitary gland may promote an increase (↑) in systemic IGF-1, a ligand of the IGF1R receptor, leading to activation of the ERK1/2 and MAPK kinases and downstream activators.

GH and IGF have essential roles in the regulation of regenerative properties of various tissues in the body, including the cornea. Human GH is a small 22 kDa protein produced primarily by the pituitary gland. During development, synchronized growth is intricately organized and driven by a number of growth factors that control cell proliferation, differentiation, and metabolism. GH secretion from the pituitary gland is known to influence tissue growth by activation of the IGF system, which is mediated by the isoforms of IGF (e.g., IGF-1 and -2), the IGF receptors (IGF-R1 and -R2), and the IGF-binding proteins that influence the binding properties of the IGF ligands. IGF-1 binding to its receptor initiates the activation of pathways associated with proliferation and survival, including the mitogen-activated protein kinase (MAPK) and ERK (extracellular signal-regulated kinase) pathways, among others (Figure 4). The members of the IGF family have collective roles in bioenergetic processes that influence cell proliferation, migration, and survival. The most studied isoform, IGF-1, has been found to have significant bioactivity in the cornea and influence corneal epithelial cell migration and proliferation with important implications in diabetic corneal disease and wound healing (reviewed in Refs. [79,80,81]).

Systemic IGF-1 levels influence not only ocular tissue growth, but glandular growth and development as well. Mouse models with varying gradients of systemic levels of GH show a two-fold increase in meibomian gland size compared to wild-type controls with GH-knockout mice exhibiting smaller, hyper-keratinized meibomian gland structure [82].

In human observational studies of conditions associated with excess GH production before or after puberty, such as gigantism or acromegaly, respectively, increased systemic levels of IGF-1 have been detected and found to be associated with an overgrowth of soft tissues. Cases of low GH availability or GH-unresponsiveness due to mutations in the GH receptor have been associated with dwarfism leading to abnormal growth patterns and reduced adult height [83,84,85]. GH treatment is a therapeutic option for both GH deficit and idiopathic short stature to promote target growth and development [86,87,88]. Low levels of IGF-1 have been correlated with reduced ocular axial length in Laron’s syndrome patients with IGF-1 supplementation reducing the disparity to normal levels [89]. Choroidal thickness, which is inversely related to ocular axial length [90], has been reported to increase at puberty depending on physical height and sexual maturation [91], with studies showing that axial length growth correlates with myopia onset [92,93]. The role of hormones in myopia development has been purported in a few older studies [94,95], but remains relatively unsupported due to the well-founded relationship of visually-guided axial length growth that can be promoted in animal models of myopia (reviewed in Ref. [96]). GH clearly plays a role during developmental tissue growth, but its effects during adulthood are still unclear.

There is some evidence suggesting positive effects of activation of the GH/IGF axis on tissue reparative responses. In studies of the skin, exogenous GH appears to promote tissue regeneration in patients and animals with severe burns leading to improved prognosis following topical application with both immediate and slow-release drug vehicles [97]. In regard to a possible mechanism, an increase in collagen deposition was observed following GH stimulation, as measured by hydroxyproline synthesis [98], showing that GH not only influences epithelial proliferation and migration, but may also affect fibroblast secretion and deposition of the ECM following injury. Consistent with these findings, the related growth hormone-releasing hormone was similarly shown to promote epithelial migration, contractility, and wound closure both in vitro and in vivo [99].

While clearly relevant to tissue regeneration, the GH/IGF-1 axis also appears to be an important signaling pathway linked to promoting matrix deposition in bone and skin. In a small cohort of older men aged 60–80 years, an increase in bone mass and skin thickness was observed following GH supplementation [100]. Similarly, GH was reported to influence corneal thickness in conditions of excess IGF-1 production post-pubescent, such as acromegaly, resulting in a ~3–7% increase in corneal thickness [101,102].

While development and certain pathological conditions are associated with high GH levels that influence tissue growth and structure, the GH/IGF axis may also be promoted via metabolic stimulation. Arginine supplementation has been purported to result in an increase in systemic GH levels [103,104,105]. As a precursor to proline, arginine has been found to promote wound healing by providing a source for the prominent amino acids required in the assembly of collagen, which is primarily composed of repeating units of proline, hydroxyproline, and glycine [106,107]. Given the mechanistic effects of arginine on GH secretion in vivo, the role of excess arginine in promoting wound healing may be related to the local effects of GH on cell proliferation and migration, in addition to localized effects of arginine on promoting collagen secretion. A recent study from our group showed that arginine supplementation promoted collagen type I secretion in vitro by primary corneal fibroblasts isolated from KC corneas, even though basal gene expression of arginase, the primary enzyme involved in arginine metabolism, was found to be elevated in these cells [108]. These studies suggest that exogenous application of key metabolites important in collagen monomer synthesis may serve to promote acute collagen deposition, which has implications in developing therapeutics to promote rapid tissue repair.

Pro-regenerative properties of GH application have been shown in cutaneous burn patients [97,109], as well as posited as a potent treatment for conditions associated with corneal epithelial defects [110]. However, whether the positive effects of topical application of GH observed in skin wounding models can be translated to the human cornea remains unclear. The intricate organization of the ECM found in the corneal stroma and the need to maintain transparency are key variants that distinguish comparisons between skin and cornea models. While GH has been patented as a potential therapeutic to increase corneal epithelial wound closure [111], prospective clinical trials are needed to assess potential therapeutic benefits of topical GH application on corneal epithelial regeneration and stromal haze development in human clinical populations, particularly in cohorts presenting with persistent corneal epithelial defects due to infection or limbal cell deficiencies. Preclinical in vitro studies have shown that exogenous GH increases corneal epithelial migration via activation of the signal transducer and activator of transcription 5 (STAT5), which is a transcription factor associated with promoting cell proliferation [112]. This work suggests that topical GH application may aid in the regeneration of the epithelium following injury. Further studies evaluating whether GH influences ECM deposition by stromal keratocytes within the stroma following wounding are needed to determine if topical GH application can blunt or prevent corneal scar development in addition to promoting recovery of the epithelium.

## 5. Conclusions

Although the cornea is avascular, it is evident that certain factors found in circulation may reach the cornea via diffusion from the tear film and/or aqueous humor. The endogenous expression of certain hormones by resident cells found within the cornea has also been posited as a local source of these factors in the anterior segment. Differential changes in androgens and estrogens during developmental growth, pregnancy, and aging may influence corneal structure, maintenance, and regeneration. While both endogenously and exogenously applied estrogens have been studied in the context of corneal biology, much less is known regarding the role of androgens in these processes. Further studies of the functional effects of androgens and estrogens in preclinical animal models are needed to extrapolate meaningful, mechanistic information from observational human clinical studies. With the widespread use of animal models in the study of human diseases, the hormone status of experimental groups should be considered when interpreting and extrapolating biological outcomes to human populations. Rather than an exclusion of groups in which hormones are likely to influence the biological outcome, a rigorous study of the role of these factors should be included within the experimental design. Since DHEA levels appear to be much lower in mice and rats than in humans, disease models utilizing rodents must take these factors into consideration when studying ocular diseases and the contribution of steroidal hormones. Further studies are required to determine the effects of hormone augmentation on corneal wound healing during physiological and pathological conditions.

## Figures and Tables

**Table 1:** Reported expression of specific hormone receptors in the primary cells of the corneal epithelium, stroma, and endothelium.

Receptor	Corneal Epithelial Cells	Corneal Stromal Fibroblasts	Corneal Endothelium	Ref.
Androgen receptor	+	+	+	[24,26,27]
Estrogen α or β receptor	+	+	+	[24,26,27]
Progesterone receptor	+	n/a	+	[24,26,27,28]
Luteinizing hormone receptor	-	+	n/a	[25]
Follicle-stimulating hormone receptor	+	+	n/a	[25]

Notations: + represents evidence supporting gene or protein expression in the respective cell type, - represents evidence supporting no gene or protein expression in the respective cell type, and n/a represents that supporting data has yet to be reported.

## Data Availability

Not applicable.
